# Disease and Disapproval: COVID-19 Concern is Related to Greater Moral Condemnation

**DOI:** 10.1177/14747049211021524

**Published:** 2021-06-10

**Authors:** Robert K. Henderson, Simone Schnall

**Affiliations:** 1 Department of Psychology, 2152 University of Cambridge, United Kingdom

**Keywords:** morality, disgust, pathogen avoidance, behavioral immune system, moral judgment, emotion, harm, COVID-19, coronavirus, moral foundations theory

## Abstract

Prior research has indicated that disease threat and disgust are associated with harsher moral condemnation. We investigated the role of a specific, highly salient health concern, namely the spread of the coronavirus, and associated COVID-19 disease, on moral disapproval. We hypothesized that individuals who report greater subjective worry about COVID-19 would be more sensitive to moral transgressions. Across three studies (*N* = 913), conducted March-May 2020 as the pandemic started to unfold in the United States, we found that individuals who were worried about contracting the infectious disease made harsher moral judgments than those who were relatively less worried. This effect was not restricted to transgressions involving purity, but extended to transgressions involving harm, fairness, authority, and loyalty, and remained when controlling for political orientation. Furthermore, for Studies 1 and 2 the effect also was robust when taking into account the contamination subscale of the Disgust Scale–Revised. These findings add to the growing literature that concrete threats to health can play a role in abstract moral considerations, supporting the notion that judgments of wrongdoing are not based on rational thought alone.

People’s moral compass is typically assumed to be firmly grounded in rational thought. For example, legal systems rely on judges and jurors making decisions about wrongdoing via detached evaluation of the available evidence. However, an emerging literature suggests that judging right or wrong can be colored by factors that are objectively unrelated to deliberate considerations, such as emotions and intuitions. Haidt (2001) proposed that such factors are the driving force behind moral judgments, with rationalizations taking place only after a decision has already been reached. Indeed, there is an increasing recognition that morality is shaped by processes that unfold largely outside of conscious awareness.

In particular, disgust has been suggested to play a role in the evaluation of moral transgressions due to its evolutionary function of disease avoidance (Rozin et al., 2008; Schnall et al., 2008; Tybur et al., 2013). Indeed, pathogens and parasites have played an outsized role in evolutionary history. For both hunter-foraging societies and our nearest evolutionary relatives, chimpanzees, about seven out of every 10 deaths can be attributed to infections (Finch, 2010, 2012). Even in armed conflict, illness has historically accounted for far more deaths than those that result from combat itself. For example, in the American civil war, two-thirds of the estimated 660,000 deaths were caused by pneumonia, typhoid, dysentery, and malaria (Connolly & Heymann, 2002). Furthermore, during famines, infectious diseases have caused more deaths than starvation as a consequence of the behavioral changes induced by conditions of extreme hunger (Shaw-Taylor, 2020). Even in modern times, nearly a quarter of all worldwide deaths have been due to infectious diseases—more than double that from violence or injury (WHO, 2015).

In light of the substantial risks posed by infectious illnesses, there has been a large body of literature investigating the associations between disease threat, behavioral caution, lower tolerance for nonconformity, and political conservatism (Mortensen et al., 2010; Murray & Schaller, 2012; Wu & Chang, 2012; Zmigrod et al., 2020). Such associations are thought to reflect the potency of disease threat, such that infectious disease concerns motivate individuals to more closely behave in line with societal expectations.

Likewise, disease threat appears to be associated with moral vigilance (Murray et al., 2019; Park & Isherwood, 2011; Van Leeuwen et al., 2012). Results from this line of research are consistent with the conceptual link between disgust and moral considerations, as disgust is thought to have evolved primarily to facilitate disease avoidance. Historically, individuals believed that violating moral proscriptions increased the likelihood of danger, and in particular the spread of infectious disease (Fabrega, 1997). Therefore, wrongdoers who violated such norms posed a threat to the survival of others. Under perilous conditions, such as during a pandemic, such norms may take on even more importance, especially to the extent that individuals subjectively evaluate the infectious disease as threatening. Indeed, Murray et al. (2019) found a positive association between sensitivity to moral wrongdoing and the germ aversion subscale of the Perceived Vulnerability to Disease scale.

Another way of approaching this issue is to examine naturally occurring concerns about physical contamination, such as the fear of contracting a highly salient contagious disease that poses an immediate threat. In other words, to explore the relationship between disease threat and morality, one can examine the relationship between concern about physical health and moral judgments directly. In early 2020, the global spread of a previously unknown type of coronavirus (SARS-CoV-2) leading to COVID-19 disease presented such an opportunity.

In March-May 2020 we assessed whether U.S. participants’ fear about catching the disease was related to their moral judgments. We did so by asking a standard polling question about coronavirus worry, and administering a set of survey items that encompassed different domains of morality. Moral Foundations Theory (Graham et al., 2009) proposes at least five moral foundations: Aversion for the suffering of others (Harm), concern with cheating and lack of reciprocity (Fairness), group adherence (Loyalty), deference to leadership and tradition (Authority), and concern with purity and contamination (Purity). These five foundations are thought to have arisen to cope with adaptive challenges in human ancestral environments. We hypothesized that individuals who report subjective worry about contracting COVID-19 would express more disapproval when evaluating wrongdoing than individuals with relatively lower worry.

## Study 1

The first study was conducted on March 17, 2020, days after COVID-19 was declared a pandemic by the World Health Organization (March 11, 2020), and a national emergency by the U.S. government (March 13, 2020). Occurrence was largely concentrated in Washington state, with 904 cases, including 48 deaths. We therefore sampled participants from this state, and as a comparison, Maine, a less densely populated state with only 17 cases and no deaths at that time. We reasoned that fear of the virus would be higher in the former than the latter state. In addition, to make the health threat salient, half the participants read a *New York Times* article on the dangers of the pandemic while the other half read a neutral article about national parks. We predicted harsher moral judgments for participants who were worried about catching the virus, compared to those who were not.

## Method

### Participants

Participants from Washington and Maine were recruited via the online participant panel Prolific. Because it was the first study, we did not have a specific effect size in mind, and aimed for a target sample of 200, collecting data from 220 participants in anticipation of possible exclusions. We removed data from 14 participants for failing attention checks. The final sample consisted of 206 participants (130 women; age: *M* = 36.80 years, *SD* = 14.16), with 165 from Washington, and 41 from Maine.^1^

### Procedure

After providing informed consent, participants were randomly assigned to read one of two *New York Times* articles, either on the dangers of coronavirus infections, or about national parks, both published on March 13, 2020. They then responded to 60 Moral Foundations Vignettes that had been pre-tested and standardized (Clifford et al., 2015). There were 12 violations for each foundation, rated on a scale from 1 (not at all wrong) to 5 (extremely wrong). Scenarios included, “You see a girl laughing when she realizes her friend’s dad is the janitor” (Harm), “You see a tenant bribing a landlord to be the first to get their apartment repainted” (Fairness), “You see a man leaving his family business to go work for their main competitor” (Loyalty), “You see a star player ignoring her coach’s order to come to the bench during a game” (Authority), and “You see two first cousins getting married to each other in an elaborate wedding” (Purity). Vignettes were administered in a randomized order.

Then participants indicated their worry about COVID-19 by responding to a standardized question taken from the popular public opinion and data company YouGov: “Taking into consideration both your risk of contracting it and the seriousness of the illness, how worried are you personally about experiencing coronavirus?” Response options included “not at all worried,” “not too worried,” “somewhat worried” and “very worried.” Participants then completed the Disgust Sensitivity Scale-Revised (Haidt et al., 1994, modified by Olatunji et al. (2007)) to assess whether the contribution of coronavirus worry went above and beyond this individual difference variable. Lastly participants provided demographics and their political orientation, rated on a scale from 1 (= very liberal) to 7 (= very conservative), and were debriefed and compensated.

## Results

### Manipulation Check

Using a 2 × 2 ANOVA we first tested whether COVID-19 case prevalence as a function of state (Washington vs. Maine) and Virus Threat Salience (Coronavirus vs. National Parks article) were associated with different levels of worry about contracting the illness. Unexpectedly, there was no effect of State, *F*(1, 202) = .74, *p* = .39, nor of Threat Salience, *F*(1, 202) = .81, *p* = .37, and no interaction, *F*(1, 202) = .03, *p* = .87, indicating that for people’s concern about the virus it did not matter as a function of whether they resided in an area with high vs. low disease prevalence, nor whether they had been primed with information about the virus, or not. We therefore were unable to analyze moral judgments based on these variables, and instead performed exploratory analyses using participants’ self-reported level of coronavirus concern.

### Moral Judgment

Following standard practice in opinion polls from which we derived the survey item (YouGov), we divided the sample into participants who were less worried (i.e., indicated they were “not at all worried” or “not too worried,” *n* = 72), and those who were more worried (i.e., indicated they were “somewhat worried” or “very worried,” *n* = 134). Furthermore, because the study was administered less than 1 week after COVID-19 was declared a national emergency in the U.S., and most participants reported at least some level of worry about COVID-19, we dichotomized the variable into “less worried” and “worried.” Dichotomizing continuous variables can be a useful approach for analyzing non-normal data (Farringdon & Loeber, 2000). Indeed, a Shapiro-Wilk test showed a significant departure from normality, W(206) = .84, *p* < .001. For each participant, the mean moral disapproval rating across the five foundations was calculated, with higher scores indicating more severe condemnation.

We then performed a repeated-measures ANOVA with Moral Foundation (Harm, Fairness, Authority, Loyalty, and Purity) as a within-subjects factor and Worry (Worried vs. Less-Worried) as a between-subjects factor. The Huynh-Feldt correction was applied because Mauchly’s test of sphericity was significant (*p* < .001). There was a main effect of moral foundation, *F*(3.47, 707.45) = 123.43, *p* < .001, η_p_^2^ = 0.38, with the highest ratings for purity (*M* = 3.61, 95% CI = [3.52, 3.70]) and fairness violations (*M* = 3.48, 95% CI = [3.40, 3.55]), followed by harm (*M* = 3.45, 95% CI = [3.36, 3.53]), authority (*M* = 3.17, 95% CI = [3.08, 3.26]), and loyalty violations (*M* = 2.78, 95% CI = [2.68, 2.89]).

**Figure 1.**
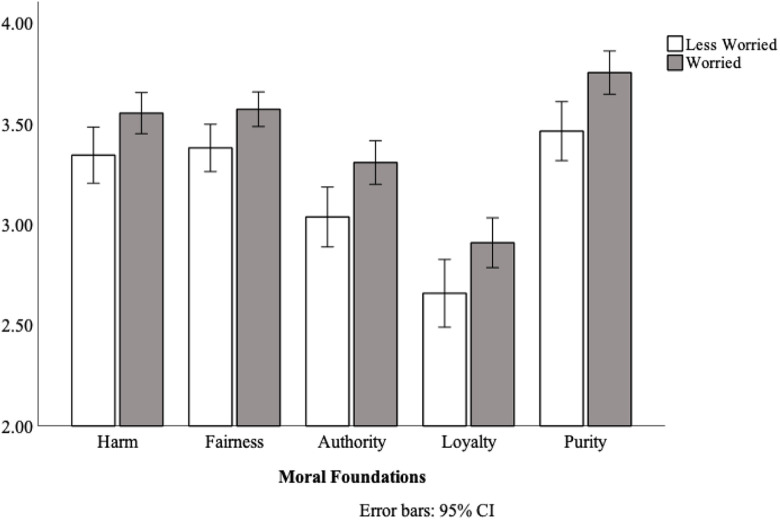
Ratings of moral condemnation for individuals who were worried, or relatively less worried about coronavirus, Study 1.

Testing the key prediction, worried participants (*M* = 3.42, *SD* = .51) produced harsher moral judgments than less-worried participants (*M* = 3.18, *SD* = .51), *F*(1, 204) = 10.66, *p* = .001, η_p_^2^ = .05 (see Figure 1 for means). Foundation Type did not interact with Worry, *F*(3.55, 724.70) = .49, *p* = .722, suggesting that the effect was comparable across foundations.

### Political Orientation

Because prior research has shown that political orientation correlates with moral judgments (e.g., Graham et al., 2009), we also conducted an ANCOVA for which we added responses to this item as a covariate to the analysis above. Consistent with earlier research, political orientation was a significant predictor of moral condemnation, *F*(1, 203) = 15.63, *p* < .001. More importantly, however, when controlling for it, the effect of coronavirus worry remained robust, *F*(1, 203) = 14.97, *p* < .001, η_p_^2^ = .069. Thus, the observed difference was not driven by political ideology.

### Contamination Disgust

Across participants, scores on the contamination subscale of the Disgust Scale–Revised (DS-R, Haidt et al., 1994, modified by Olatunji et al., 2007) was associated with COVID-19 worry (*r* = .20, *p* < .001). To investigate the possible effects of contamination disgust on moral judgment between the two groups, a repeated-measures ANCOVA was performed with contamination disgust as a covariate. There was an effect for this covariate, *F*(1, 203) = 22.10, *p* < .001, η_p_^2^ = .10. More importantly, however, the main effect of coronavirus worry remained significant after controlling for contamination disgust, *F*(1, 203) = 6.28, *p* = .013, η_p_^2^ = .03.

## Discussion

Study 1 provided initial evidence that the extent to which people were worried about coronavirus in the early days of the 2020 COVID-19 pandemic related to condemnation of moral transgressions that were unrelated to the virus: Participants who were worried about COVID-19 produced harsher moral judgments than those who were less worried. Importantly, this effect could not be attributed to political orientation, consistent with earlier findings that the role of disgust in moral judgment is not explained by ideology (van Leeuven et al., 2017). We furthermore controlled for contamination disgust, and although it accounted for some of the variance, the association between worry and moral judgment remained robust.

However, we had included a threat salience manipulation at the beginning of the study, which turned out to be ineffective, and considered it important to replicate the effect without such a procedure before participants made the moral judgments. We therefore conducted another study, and as additional improvement also counterbalanced the order in which the moral judgments and the coronavirus worry question were administered.

## Study 2

The second data collection was carried out 10 days later, on March 27, 2020, when COVID-19 cases across the U.S. had risen somewhat but were still relatively localized, with 3,700 cases in Washington state, including 174 deaths, but only 168 cases and one death in Maine, respectively. We again focused on these two states as representing objectively different virus threats, in case this was important for subjectively experienced worry about the virus.

## Method

### Participants

We recruited participants via Amazon Mechanical Turk from Washington and Maine. Building on the observed effect size from Study 1, *d* = .47, a G*Power analysis (Faul et al., 2007) indicated a required sample size of 238 for an independent samples t-test (two-tailed) with 95% power at α = .05. We removed data from 11 participants because they did not complete the study, and from seven participants for failing attention checks. The final sample consisted of 220 participants (126 women; age: *M* = 39.11 years, *SD* = 12.69), with 189 from Washington, and 44 from Maine.

### Procedure

Identical materials, measures and procedure as in Study 1 were used, but there was no coronavirus manipulation at the beginning, and administration of the moral stimuli and the coronavirus worry question was counterbalanced.

## Results

### Manipulation Check

We first conducted a one-way ANOVA to test whether coronavirus worry differed between residents of Washington and Maine. Consistent with the results from Study 1, there was no effect, *F*(1, 218) = .002, *p* = .97. We therefore used the same analysis strategy and focused on participants’ self-reported level of coronavirus concern (i.e., their subjectively experienced threat).

### Moral Judgment

We performed a repeated-measures ANOVA with Moral Foundations (Harm, Fairness, Authority, Loyalty, and Purity) as a within-subjects factor and Worry (Worried vs. Less-Worried) and Order (Worry Question first vs. Moral Judgments first) as between-subjects factors. The Huynh-Feldt correction was used because Mauchly’s test of sphericity was significant (*p* = .001). Results revealed no main effects of order *F*(1,216) = 0.12, *p* = .725, nor any Order × Group interaction, *F*(1, 216) = 0.22, *p* = .637. Therefore, order was not further considered.

**Figure 2.**
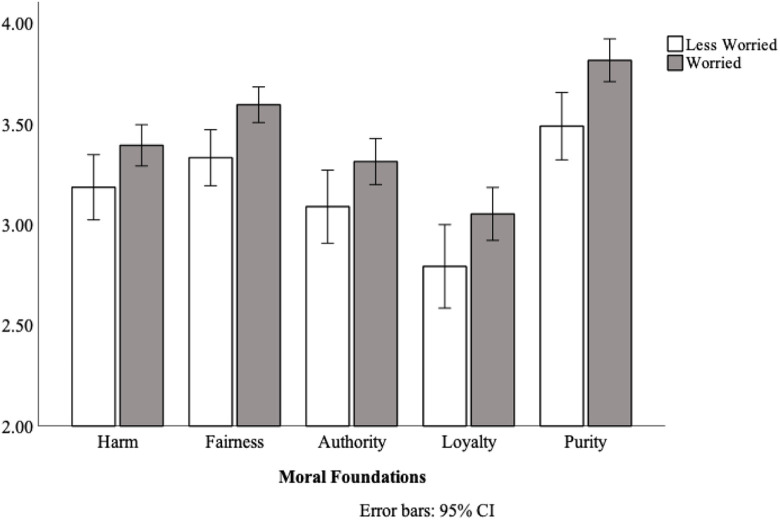
Ratings of moral condemnation for individuals who were worried, or relatively less worried about coronavirus, Study 2.

There was a main effect of moral foundation, *F*(3.48, 757.52) = 75.602, *p* < .001, η_p_^2^ = 0.26, with the highest ratings for purity violations (*M* = 3.65, 95% CI = [3.55, 3.75]), then fairness (*M* = 3.46, 95% CI = [3.38, 3.55]), followed by harm (*M* = 3.29, 95% CI = [3.19, 3.39]), authority (*M* = 3.20, 95% CI = [3.09, 3.31]), and loyalty violations (*M* = 2.92, 95% CI = [2.80, 3.05]). Replicating the results of Study 1, there was a main effect of worry, such that worried participants (*M* = 3.43, *SD* = .58) showed greater moral condemnation than less-worried participants (*M* = 3.18, *SD* = .60), *F*(1, 218) = 8.67, *p* = .004, η_p_^2^ = .04 (see Figure 2). There was no foundation × worry interaction, *F*(3.48, 757.52) = .52, *p* = .694, again indicating that the effect was not limited to any specific foundations, such as purity.

### Political Orientation

To rule out this possible confound, the same analysis as above was conducted with political orientation as a covariate. Consistent with earlier research, political orientation was a significant predictor of moral condemnation, *F*(1, 217) = 29.64, *p* < .001. As was the case for Study 1, there still was a significant difference between the worried and less-worried participants for moral disapproval, *F*(1, 217) = 13.47, *p* < .001, η_p_^2^ = .058, again showing the independent contribution of disease concern.

### Contamination Disgust

Consistent with Study 1, contamination disgust was associated with COVID-19 worry (*r* = .20, *p* = .003). To again investigate the possible effects of contamination disgust on moral judgment between the two groups, a repeated-measures ANCOVA was performed with scores on the contamination subscale of the Disgust Scale-Revised (Olatunji et al., 2007) as a covariate. Contamination disgust showed a significant effect regarding moral condemnation, *F*(1, 217) = 54.10, *p* < .001, η_p_^2^ = .20. Importantly, and replicating the results from Study 1, the effect of coronavirus worry on moral judgment remained significant after controlling for contamination disgust, *F*(1, 217) = 4.22, *p* = .041, η_p_^2^ = .02.

## Discussion

This study replicated the observation that a situational threat to one’s physical health, namely concern about contracting an illness that was spreading rapidly throughout the U.S. at the time, was related to moral concerns. Speaking to ongoing debates of whether the link between disgust and morality is domain-specific, or more general (see Schnall, 2017, for a discussion), in both studies the effect was not specific to transgressions involving purity, but extended to all moral foundations.

## Study 3

Both studies 1 and 2 included samples from only two areas of the U.S. that had varying levels of cases of COVID-19. Participants in these states did not differ in subjectively perceived worry about the virus, thus justifying the use of the latter as the predictor variable. These findings still raise the question, however, of whether the same effect would be observed more broadly across the population. In particular, while in mid-to-late March 2020, when the first two studies were conducted, COVID-19 cases were relatively low in the U.S., it was also important to explore whether the effects would persist as the pandemic unfolded across the country. We therefore conducted a preregistered replication about 6 weeks after Study 2, sampling across the entire U.S. On May 6, 2020, when the study was conducted, there were 1,261,354 COVID-19 cases, including 74,710 deaths. We again predicted that people worried about the virus would rate moral infractions as more objectionable than those who were less worried.

## Method

### Participants

Participants were recruited across the U.S. with Prolific. Using the effect size from Study 2, *d* = .42, a G*Power analysis using 95% power at α = .05 specified a required sample of 296 participants. However, because the earlier studies were conducted only in Maine and Washington, to account for increased variability when sampling across the entire population of the U.S., we set our preregistered sample to 500. Data from 13 participants were excluded because they failed attention checks. The final sample involved 487 participants (273 women; age: *M* = 31.25, *SD* = 11.77).

### Procedure

The method was identical to Study 2.

## Results

We performed a repeated-measures ANOVA with Moral Foundations (Harm, Fairness, Authority, Loyalty, and Purity) as a within-subjects factor and Worry (Worried vs. Less-Worried) and Order (coronavirus question first or moral foundations vignettes first) as between-subjects factors. The Huynh-Feldt correction was used as Mauchly’s test of sphericity was significant (*p* < .001). Results revealed no main effects of Order *F*(1,483) = 0.85, *p* = .356, and no Order × Group interaction, *F*(1, 483) = 1.79, *p* = .182. Therefore, order was not further considered.

Replicating the earlier results, there was a main effect of moral foundation, *F*(3.37, 1631.87) = 186.20, *p* < .001, η_p_^2^ = 0.28, with the highest ratings for Purity violations (*M* = 3.70, 95% CI = [3.63, 3.76]), followed by fairness (*M* = 3.49, 95% CI = [3.43, 3.56]), harm (*M* = 3.45, 95% CI = [3.39, 3.52]), authority (*M* = 3.26, 95% CI = [3.19, 3.33]), and loyalty violations (*M* = 2.88, 95% CI = [2.80, 2.96]).

**Figure 3.**
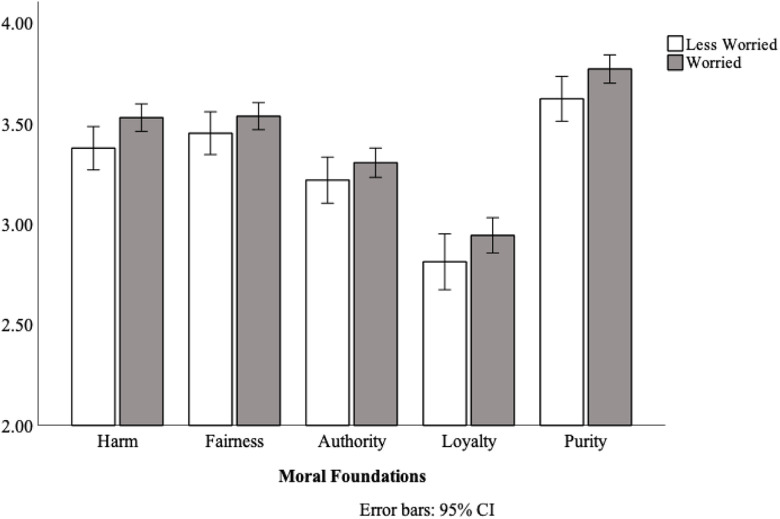
Ratings of moral condemnation for individuals who were worried, or relatively less worried about coronavirus, Study 3.

Consistent with the findings from Studies 1 and 2, and as specified in our preregistration (https://aspredicted.org/blind.php?x=2as8ic), worried participants (*M* = 3.42, *SD* = .59) exhibited harsher moral judgments than less-worried participants (*M* = 3.30, SD = .52), *F*(1, 485) = 4.43, *p* = .036, η_p_^2^ = .01 (see Figure 3). There was no Foundation × Worry interaction, *F*(3.37,1631.87) = .53, *p* = .686. Thus, as was the case for Studies 1 and 2, the specific moral foundation did not moderate the effect of coronavirus worry on moral judgments.

### Political Orientation

To once again test for possible effects of political orientation on moral judgment between the two groups, a repeated-measures ANCOVA was performed as before. As with Studies 1 and 2, political orientation had a significant effect on moral condemnation, F(1,484) = 29.50, p < .001. Also replicating the earlier findings, there was a statistically significant difference between the worried and less-worried participants for moral disapproval, *F*(1, 484) = 5.82, *p* = .016, η_p_^2^ = .01, such that worried participants rated moral violations as more objectionable than less-worried participants. Thus, political orientation was not a confound.

### Contamination Disgust

Contamination disgust was associated with COVID-19 worry (*r* = .30, *p* < .001), which is consistent with Studies 1 and 2. Following the logic of the earlier studies, we investigated the possible contribution of contamination disgust on moral judgment by performing another analysis that included the contamination subscale of the DS-R as a covariate. This variable was again significantly related to moral condemnation, *F*(1, 484) = 77.50, *p* < .001, ηp^2^ = .14. In contrast to the earlier two studies, however there was no longer a significant effect for coronavirus worry on moral judgment after controlling for the effect of contamination disgust, *F*(1, 484) = .005, *p* = .944, ηp^2^ = .00, indicating that contamination concerns were largely responsible for the effect descried above.

### Contamination Disgust Over Time

Because the finding that the relationship between worry and moral judgments was no longer significant after controlling for contamination disgust, we considered potential reasons for this unexpected result. One possibility is that contamination scores increased over time, as people became increasingly familiar with the coronavirus threat. Indeed, the effect size of the association between contamination disgust and COVID-19 worry was identical in Studies 1 and 2, but it was larger in Study 3. We therefore tested whether the participants in May reported higher contamination disgust relative to the participants tested in March. A one-way ANOVA was conducted to determine if contamination disgust scores were different between Studies 1, 2, and 3. There was a main effect of contamination disgust across the studies, *F*(2, 910) = 9.63, *p* < .001, η^2^ = .02. Tukey post hoc tests revealed that contamination disgust in Study 1 (*M* = 2.63, *SD* = .78) and Study 2 (*M* = 2.68, *SD* = .87) did not differ, *p* = .792 (.05, 95% CI [−.13, .24]). However, the mean increase between Study 1 and Study 3 (*M* = 2.89, *SD* = .81) was significant (.26, 95% CI [.10, .42], *p* < .001), as was the mean increase between Study 2 and Study 3 (.21, 95% CI [.05, .37], *p* = .004). Thus, while there was no significant increase between the March 17 and March 27 groups, there was a significant rise in contamination disgust between the March groups and the May 6 group, suggesting that as the pandemic wore on, people may have become more sensitive to contamination, and that this concern therefore overshadowed the contribution of coronavirus worry alone.

## Discussion

This preregistered study largely replicated the findings from Studies 1 and 2. What is noteworthy, however, is that the magnitude of the effect was somewhat smaller. One possibility is the fact that the sample was more diverse in many respects, given that it came from all across the U.S. In addition, it might have also mattered that nearly 2 months had passed since the outbreak of the pandemic, with many areas having issued stay-at-home orders by that point. Indeed, inspecting the means revealed that, while moral judgments among worried participants were practically identical across the three studies (Study 1: *M* = 3.42; Study 2: *M* = 3.43; Study 3: *M* = 3.43), the moral judgments for less worried participants were higher in Study 3 (*M* = 3.30) than in Study 1 (*M* = 3.18) and Study 2 (*M* = 3.18). An exploratory ANOVA comparing this group in March vs. May found a marginally significant effect, *F*(1, 272) = 3.42, *p* = .066, *d* = .22, such that participants in May who were relatively less worried about the disease produced harsher moral judgments than participants less worried about the disease in March, perhaps as a function of extended exposure to this threat. Lastly, we found that the association between worry and moral condemnation was no longer significant when controlling for contamination disgust, indicating that the fear of pathogens was largely responsible for the effect of COVID-19 worry on moral condemnation.

## Internal Meta-Analysis

Since the methods were largely the same across the three studies, we combined the data sets (*N* = 913) to conduct a mini meta-analysis, following the recent best-practices recommendations of a number of researchers and statisticians (e.g., Goh et al., 2016; Lakens & Etz, 2017; McShane & Böckenholt, 2017). Based on the guidelines proposed by Goh et al. (2016), we used fixed effects in which the effect sizes within each study and the mean effect sizes across the three studies were weighted by sample size. We first converted Cohen’s *d* effect sizes (Study 1: *d* = .47, Study 2: *d* = .42, Study 3: *d* = .24) into Pearson’s *r* for ease of analysis. All effect sizes were then Fisher’s *z* transformed for analyses and converted back to Pearson’s *r* for presentation. Overall, the effect was significant, *M_r_* = .15, *Z* = 4.81, *p* < .001, two-tailed, such that individuals worried about COVID-19 rated moral violations as more objectionable than those who were less worried. A fully random effects test of the overall effect was also significant, as indicated by a one-sample t-test of the mean effect size against zero, *M_r_* = .17, *t*(2) = 4.67, *p* = .043, two-tailed.

Although all three studies showed no moderating role of foundation type, we nevertheless considered it instructive to explore such potential differences, given the research interest that the question of specificity to moral domain has received. Purity (*M_r_* = .16, *p* < .001) showed the strongest effect, which makes sense given that COVID-19 poses a direct threat to one’s physical health. Significant effects, however, also occurred for Harm (*M_r_* = .13, *p* < .001), Fairness (*M_r_* = .12, *p* < .001) Authority (*M_r_* = .11, *p* < .001) and Loyalty (*M_r_* = .11, *p* < .001), suggesting that the effect was relatively broad. A fully random effects test of the effects against zero yielded significant results for three of the five moral foundations as indicated by one-sample t-tests against zero (all two-tailed). The strongest effect was for Purity, *Mr* = .18, *t*(2) = 5.13, *p* = .036, followed by Harm, *M r* = .14, *t*(2) = 11.94, *p* = .01 and Authority, *Mr* = .12, *t*(2) = 4.52, *p* = .046. The remaining two foundations, Fairness, *M r* = .15, *t*(2) = 3.38, *p* = .077, and Loyalty, *M r* = .13, *t*(2) = 3.28, *p* = .081, reached marginal significance. Thus, the meta-analysis revealed a small-to-medium sized effect (Funder & Ozer, 2019) of worry about the coronavirus on moral condemnation across different content domains.

## General Discussion

This research tested the role of situational concerns about an infectious disease on judgments of wrongdoing. Across three studies we consistently found that people who were worried about COVID-19 condemned moral wrongdoers more harshly than those who were less worried. This finding adds to emerging work on the role of disease threat on moral judgment. In Studies 1 and 2 controlling for individual differences in contamination disgust left the effect of coronavirus worry and moral judgment intact. In contrast, in Study 3, we found that this relationship was no longer significant after accounting for contamination disgust, indicating that fear of contamination was responsible for the effect. We interpret this finding to be the result of a generally heightened concern about the virus at the time. Indeed, contamination disgust has been described as bearing a “striking similarity” to disease avoidance (Olatunji et al., 2009). An intriguing possibility is, therefore, that variables that are typically considered to reflect stable individual differences, such as disgust sensitivity, may change as a function of coronavirus concerns that became relatively universal across the world. Indeed, recent theorizing has suggested that topics within the field of of psychology, and the scientific approaches to study them, may change in the wake of the COVID-19 pandemic (Rosenfeld et al., in press). Given the current findings, apart from contamination and disease concerns, other relevant traits such as neuroticism or conscientiousness may also have changed over the course of the pandemic as a function of constantly having been engaged in disease-prevention behavior to alleviate related worries. Future research would be needed to explore this possibility.

Our findings align with a growing body of research demonstrating that individual differences in the propensity to experience disgust are linked to moral considerations (Chapman & Anderson, 2014; Karinen & Chapman, 2019; Liuzza et al., 2019; Murray et al., 2019; Robinson et al., 2019; Wagemans et al., 2018). Furthermore, the results are consistent with recent work showing a positive association between germ aversion and moral condemnation across the moral foundations (Murray et al., 2019). Our findings contribute to this line of research by demonstrating that subjective worry about a real-world contagious disease is associated with harsher moral judgments, and, moreover, that this relationship held even after accounting for differences in political orientation. Thus, converging evidence supports Haidt’s (2001) suggestion that morality is shaped by various emotions and intuitions, of which concerns about health and safety are prominent.

There are limitations within these findings. Though we obtained large samples with consistent results across all three studies, we used a single item to measure “worry,” which may have reduced sensitivity in capturing participants’ level of concern about COVID-19. Another qualification to these results is the difference in the relationships between the trait-like measures of COVID-19 worry and moral judgments, and the effects of the experimental manipulation in Study 1. That is, although dispositional worry about contracting the illness was consistently related to moral condemnation, experimentally manipulating the salience of COVID-19 had no effect on moral judgment, relative to a neutral condition. One possibility for why is by the time of Study 1 on March 17, news about COVID-19 was already highly salient, and thus the experimental manipulation did not have the intended effect. The dispositional association, however, might be explained by a generalized overreaction to potential harm. It is possible that those who are prone to chronic worry about contracting an infectious illness are also more sensitive to moral violations in disease-relevant domains as well as other moral infractions. That is, fear of disease may overlap with an overgeneralized reaction of increased sensitivity to potential harm, including moral wrongdoers who commit not only purity violations, but other unfavorable acts as well. Indeed, worried participants produced harsher judgments than less worried participants, and there was no moderating effect of moral foundation. This is consistent with previous research, indicating that disease threat concerns are associated with conformity to moral proscriptions that are not specific to disease (e.g., Murray et al., 2011; Tybur et al., 2016; Wu & Chang, 2012). Lack of moderation by foundation type is likewise consistent with error management, such that the more costly error is to be under-vigilant about moral violations that are not disease relevant than to be over-vigilant solely for disease-relevant violations (Haselton et al., 2015; Murray et al., 2019). Further research is needed to more carefully explore these dispositional versus experimental differences.

Additionally, we did not test whether other variables, such as personality, might have played a role in our results. Disease avoidance has been associated with both neuroticism and conscientiousness (Oosterhoff et al., 2018), while openness, conscientiousness, and agreeableness have been associated with sensitivity to moral violations (Hirsh et al., 2010; Smillie et al., 2020). Thus, considering the overlap between disease avoidance, moral judgments, and conscientiousness, this personality trait may account for some of the variance between worry about a highly salient communicable disease and assessments of moral wrongdoing.

Our research raises the possibility that during a period of widespread concern about infectious disease, people may become more judgmental overall. In other words, people’s actions and intentions might be under more scrutiny, and when ambiguous, may be interpreted uncharitably, potentially resulting in misunderstandings, or interpersonal conflicts. Indeed, in the early days of the unfolding COVID-19 crisis, there were media accounts of mistrust in public officials, the press, and health organizations. The current findings suggest that we may see further instances of uncharitable evaluations as people are especially concerned for their physical health. Thus, the ongoing pandemic presented an ecologically relevant way of examining the role of disease prevalence on an issue of critical applied importance.

## Supplemental Material

Supplemental Material, sj-pdf-1-evp-10.1177_14747049211021524 - Disease and Disapproval: COVID-19 Concern is Related to Greater Moral Condemnation Click here for additional data file. Supplemental Material, sj-pdf-1-evp-10.1177_14747049211021524 for Disease and Disapproval: COVID-19 Concern is Related to Greater Moral Condemnation by Robert K. Henderson and Simone Schnall in Evolutionary Psychology

## Notes

1 We had not anticipated the Maine sample to be much smaller, but in hindsight, it is likely that fewer people were signed up on Prolific than in Washington. As noted, the states did not differ regarding coronavirus worry, and we analyzed the combined sample, so this was no longer an issue.
