# Supplementary material for: Disease and Disapproval: COVID-19 Concern is Related to Greater Moral Condemnation
Source: Evol Psychol. 2021 Jun 10;19(2):14747049211021524. doi: 10.1177/14747049211021524 (PMC10358411; doi:10.1177/14747049211021524)
Supplement: Supplemental Material, sj-pdf-1-evp-10.1177_14747049211021524 - Disease and Disapproval: COVID-19 Concern is Related to Greater Moral Condemnation [file sj-pdf-1-evp-10.1177_14747049211021524.pdf]

Supplementary Information

Since a main objective of the current investigation was to shed light on the emerging relationship between infectious illness and moral judgment, we also conducted further analyses involving the Disgust Sensitivity-Revised scale (DS-R, Haidt et al., 1994, modified by Olatunji et al., 2007), which was administed at the end of each study. As would be expected, it was correlated with coronavirus worry, with $r(206) = .22$, $p = .002$ in Study 1, $r(220) = .27$, $p < .001$ in Study 2, and $r(487) = .27$, $p < .001$ in Study 3, respectively, although these correlations were not particularly high. More importantly, in each study there was a medium-sized association between disgust sensitivity and moral judgments (see Table 1), therefore replicating earlier research documenting this effect (Chapman & Anderson, 2014; Karinen & Chapman, 2019).

Table 1: Correlations between disgust sensitivity and moral condemnation

	Study 1	Study 2	Study 3
Overall	.44**	.51**	.41**
Harm	.28**	.34**	.31**
Fairness	.26**	.34**	.24**
Loyalty	.40**	.48**	.41**
Authority	.34**	.41**	.34**
Purity	.51**	.57**	.35**

** Correlation is significant at the 0.01 level (two-tailed).

$N = 206$ (Study 1), $N = 220$ (Study 2), $N = 487$ (Study 3)

In contrast to some previous work (Wagemans, Brandt, & Zeelenberg, 2018), however, the correlations were statistically significant, and of mostly comparable magnitude

across foundations, rather than being specific to the purity foundation. One methodological difference was that in our studies, and the ones reported by Chapman and Anderson (2014) and Karinen and Chapman (2019) the moral judgments came first, and the DS-R later, whereas Wagemans et al. (2018) used the reverse order, which might have primed participants with concepts of disgust, or induced such feelings. However, the general question of whether only purity violations are related to disgust and the behavioral immune system is complex, and awaits further investigation (see Schnall, 2017, and Wagemans et al., 2018, for discussions of the issue). Nevertheless, although secondary to the overall objective of the research of testing the link between situational disease threat and moral condemnation, these findings fit with the broader notion that disgust and morality share a common mechanism.
